# Perioperative Immune-Checkpoint Inhibition in Renal Cell Carcinoma: Lessons Learned and Future Directions

**DOI:** 10.3390/cancers18030527

**Published:** 2026-02-06

**Authors:** Adam Adika, Matthew Zibelman

**Affiliations:** Fox Chase Cancer Center, Temple Health, Philadelphia, PA 19111, USA; matthew.zibelman@fccc.edu

**Keywords:** perioperative, immunotherapy, immune-checkpoint inhibition (ICI), renal cell carcinoma (RCC), neoadjuvant, adjuvant, KIM-1, artificial intelligence

## Abstract

**Background/Objectives**: Localized renal cell carcinoma (RCC) unfortunately has a suboptimal rate of metastatic recurrence. Immune-checkpoint inhibition (ICI), commonly known as immunotherapy, is being increasingly utilized in the management of other localized cancers. The objective of this paper is to review the most recent literature as it pertains to ICI in localized RCC, highlight major lessons learned across clinical trials, and then suggest new directions based on these lessons and on new technologies. **Methods**: English-language articles were identified in PubMed/Google Scholar/Scopus. Selection priority was placed on prospective clinical trials. Particular emphasis was placed on disease-free survival (DFS), overall survival (OS), and clinical trial design. Novel approaches to the diagnosis, risk stratification, and management of RCC were also queried. **Results**: The KEYNOTE-564 trial demonstrated DFS and OS benefits of adjuvant pembrolizumab in high-risk clear cell RCC. Although discordance exists with adjuvant ICI in the IMmotion-010 and CheckMate-914 trials, preliminary data from the RAMPART trial also reveal a DFS benefit for adjuvant durvalumab–tremelimumab. The PROSPER trial failed to show a benefit for perioperative nivolumab, but ongoing neoadjuvant/perioperative ICI trials show promise. Subtle differences in design exist across the mentioned trials and are arguably clinically significant. Several new diagnostic tools and technologies have been identified for use in RCC. **Conclusions**: By applying the lessons learned from the differences in clinal trial design, along with leveraging new diagnostic tools and technologies, there is great potential to more accurately stratify risk amongst patients with localized RCC and therefore better identify the patients who are most likely to benefit from perioperative ICI.

## Simple Summary

Historically, localized renal cell carcinoma has been typically treated with surgery alone. However, it is estimated that up to 30% of these patients will later develop metastatic disease. A key question in the field has been how to reduce this rate of metastasis. In recent years, neoadjuvant and/or adjuvant immune-checkpoint inhibition (referred to interchangeably as immunotherapy) has gained traction in the management of several types of cancer. The aim of this article is to review the most recent literature corresponding to immune-checkpoint inhibition in the localized renal cell carcinoma arena, highlight the major lessons learned, and suggest new directions for future investigation.

## 1. Introduction

Over 400,000 cases of renal cell carcinoma (RCC) were estimated to be diagnosed worldwide in 2022 [1]. In the United States, it is classified as the seventh most common type of cancer as of 2025 [2]. A total of 70–80% of cases are of the clear cell histological subtype [3]. In the localized setting, the standard approach for definitive treatment has been surgery, including radical or partial nephrectomy. However, studies suggest that up to 30% of patients undergoing definitive surgery will ultimately develop metastatic disease [4]. Thus, perioperative therapy with the objective of reducing the risk of recurrence of RCC and ultimately improving overall survival (OS) has been of great interest.

In recent years, immune-checkpoint inhibition (ICI) has become the standard of care in advanced RCC with the CheckMate-214 trial demonstrating an OS benefit for ipilimumab (CTLA-4) plus nivolumab (PD-1) compared to sunitinib among intermediate- and poor-risk patients with untreated advanced RCC [5]. Subsequently, three separate trials (KEYNOTE-426, CLEAR, CheckMate 9ER) revealed an OS benefit combining vascular endothelial growth factor tyrosine kinase inhibitor (VEGF-TKI) to PD-1 inhibition [5,6,7]. Thus, ICI plus VEGF-TKI was brought into the first-line setting for advanced RCC. However, few patients are considered to have curable disease with these therapies and several mechanisms of resistance to ICI and VEGF-TKI have been elucidated in the metastatic setting [8]. This naturally leads to the question of whether providing ICI earlier on in the RCC disease course can help reduce the risk of disease recurrence. Therefore, the aim of this review article is to provide insight into this question by analyzing the current literature and putting the data into perspective.

## 2. Materials and Methods

A narrative review was performed with a focus on ICI administration in localized, high-risk clear cell renal cell carcinoma (ccRCC). This included ICI in the neoadjuvant and/or adjuvant setting. English-language articles were identified in PubMed, Google Scholar, and Scopus. Selection priority was placed on prospective clinical trials. Particular emphasis was placed on disease-free survival (DFS), overall survival (OS), and clinical trial design. Novel approaches to the diagnosis, risk stratification, and management of RCC were also queried. The literature was reviewed between July 2025 and November 2025. Studies published through November 2025 were eligible. Although no specific inclusion and exclusion criteria were set, selection preference was given to randomized phase III prospective clinical trials involving perioperative ICI according to their perceived impact on actual clinical practice. This includes highly cited trials with relatively large sample sizes and publications in peer-reviewed journals with high impact factors. It must be stated that a limitation of this is that this is narrative review.

## 3. Results

### 3.1. Role of Adjuvant Immunotherapy

Historically, multiple adjuvant trials (ASSURE, ATLAS, and PROTECT) failed to show the efficacy for adjuvant VEGF-TKI therapy [9,10,11]. One exception was the S-TRAC trial, which showed a benefit in disease-free survival of 6.8 years (95% CI, 5.8-NR) vs. 5.6 years (95% CI, 3.8–6.6) when adjuvant sunitinib was compared with the placebo in patients at high risk of RCC recurrence after nephrectomy. However, no overall survival benefit was found, and sunitinib was not widely adopted. Based on treatment patterns for metastatic RCC, the failure of adjuvant VEGF-TKI led to the investigation of adjuvant immunotherapy.

The KEYNOTE-564 trial, first published in 2021, is currently the landmark trial for adjuvant immunotherapy in resected ccRCC. This phase 3, double-blind, randomized, multi-center trial investigated one year of pembrolizumab vs. placebo in 994 patients with clear cell renal cell carcinoma at high risk for recurrence after nephrectomy (with or without metastectomy). High risk of recurrence was defined as tumor (T) stage 2 (by TNM grading) with nuclear grade 4 or sarcomatoid differentiation, T stage 3 or higher with any grade, regional lymph-node metastasis, or M1 with no evidence of disease (NED). Stage M1 with NED was defined as metastases resected at nephrectomy or within 1 year of nephrectomy. At a median of 24.1 months, disease-free survival was 77.3% in the pembrolizumab group vs. 68.1% in the placebo group (hazard ratio for recurrence or death, 0.68; 95% confidence interval [CI], 0.53 to 0.87; *p* = 0.002). At a median of 48 months, significant improvement in overall survival was observed with pembrolizumab as compared with placebo (hazard ratio for death, 0.62; 95% CI, 0.44 to 0.87; *p* = 0.005). The estimated overall survival at 48 months was 91.2% in the pembrolizumab group as compared with 86.0% in the placebo group [12].

The results of KEYNOTE-564 cemented adjuvant pembrolizumab as the standard of care in high-risk ccRCC. Of note, only 48% of patients who experienced cancer recurrence in the placebo arm subsequently received anti-PD-1 or anti-PD-L1 therapy (69% received any type of subsequent anticancer therapy). It is thus unclear as to whether the overall survival benefit seen is due to ICI given specifically in the adjuvant setting or due to ICI given at any point in the disease course. This presents a source of confounding and could potentially explain the relatively modest overall survival benefit found in the study.

The IMmotion-010 trial, published in 2022, was a phase 3, double-blind, randomized, multi-center trial investigating one year of atezolizumab vs. placebo in 778 patients with renal cell carcinoma with a clear cell or sarcomatoid component and increased risk of recurrence after nephrectomy (with or without metastectomy). Approximately 8% of patients did not have clear cell histology. Increased risk was defined as stage T2 with Fuhrman grade 4, stage T3a with grade 3–4, T3b–c with any grade, T4 with any grade, or TxN+ with any grade. The M1 no evidence of disease (NED) category included patients with synchronous metastatic disease to the adrenal gland or lung, or metachronous metastatic disease to the lung, lymph node, or soft tissue, with recurrence occurring more than 12 months following initial nephrectomy. Approximately 65% of patients had T3a disease or lower. At a median follow up of 45 months, no statistically significant difference was found in disease-free survival amongst the adjuvant atezolizumab vs. placebo groups. A statistically significant difference for overall survival was also not found among the groups [13].

The CheckMate-914 trial, first published in 2023, was a phase 3, double-blind, randomized, multi-center trial of adjuvant nivolumab plus ipilimumab vs. placebo (part A) and adjuvant nivolumab monotherapy vs. ipilimumab–nivolumab vs. placebo (part B) in patients with localized RCC at high risk of relapse after partial or radical nephrectomy. High risk was defined as pathological TNM staging pT2a (grade III–IV) N0M0, pT2b (any grade) N0M0, pT3 (any grade) N0M0, pT4 (any grade) N0M0, or pT any (any grade) N1M0. M1 disease was not included, but only 15% and 12% of patients had T2 disease in parts A and B, respectively. Patients received only 24 weeks of adjuvant treatment or placebo. At a median follow up of 37 months in part A and 27 months in part B, no statistically significant difference in disease-free survival was found. Overall survival data were not mature at the time of analysis. Strikingly, only 57% and 58% of patients fully completed nivolumab plus ipilimumab treatment in parts A and B, respectively [14,15]. Hence, the large proportion of patients who did not complete treatment may have impacted the outcomes.

A central question in the RCC arena is the discordance between the positive results in the efficacy of KEYNOTE-564 and the negative results in the efficacy of IMmotion-010 and CheckMate-914. While likely multifactorial, subtle differences in study designs and accrued populations may account for the different outcomes. IMmotion-010 allowed non-clear cell histology (as opposed to KEYNOTE-564) and M1 NED (Metastatic 1, no evidence of disease) was defined as metachronous metastatic disease (to lung, lymph node, or soft tissue) with recurrence occurring more than 12 months following initial nephrectomy (in contrast to within one year following nephrectomy in KEYNOTE-564). Thus, one may argue that IMmotion-010 allowed more indolent M1 disease patients, and, given that the fraction of these patients was higher in IMmotion-010 (14% vs. 6%), a small but relevant effect on survival may have existed. Regarding CheckMate-914, one striking point is that treatment was only planned for approximately 6 months (compared to 12 months in KEYNOTE-564). Moreover, high rates of toxicity resulted in high rates of ICI discontinuation, further widening the difference in treatment duration amongst the two studies. As a result, less treatment may have contributed to the discordant results compared to KEYNOTE-564.

Recently, in October of 2025, a press release was published about LITESPARK-022, a phase 3 trial investigating adjuvant belzutifan (oral hypoxia-inducible factor-2 alpha (HIF-2-alpha) inhibitor) with or without pembrolizumab in patients with ccRCC following nephrectomy. Per the report, the combination of belzutifan and pembrolizumab met the study’s primary endpoint with a DFS benefit compared to pembrolizumab plus placebo. The results are scheduled to be released at a future medical meeting. Information regarding overall survival, a key secondary endpoint, is still pending [16].

### 3.2. Neoadjuvant/Perioperative Immunotherapy

In recent years, the use of neoadjuvant/perioperative immunotherapy-based treatment has gained traction in multiple disease sites. It has demonstrated efficacy in prospective clinical trials in melanoma [17], non-small cell lung cancer [18], breast cancer [19], and bladder cancer [20]. Several potential advantages have been posited to account for the benefits of these approaches. One possible explanation may be that starting treatment in the presence of primary tumors may result in increased tumor antigen load and diversity. This can then be leveraged to expose greater tumor targets to the immune system, creating a more robust and durable immune response. This response can then also be utilized to attack micrometastatic cancer cells, possibly even after definitive surgery. Further, tumor shrinkage may allow for better surgical outcomes. Finally, the pathology of the surgical specimen may be useful as an early surrogate for efficacy. However, despite the efficacy seen in other cancers and the proposed mechanisms of this success, progress in the neoadjuvant arena of RCC has stalled.

A landmark trial in the RCC neoadjuvant space is the EA8143 (PROSPER) trial. It was a phase 3, open-label, randomized trial comparing perioperative nivolumab with surgery alone in patients with intermediate-to-high risk of recurrence renal cell carcinoma. A total of 819 patients across 183 sites in the USA and Canada were randomized in 1:1 ratio to surgery followed by surveillance vs. neoadjuvant nivolumab (480 mg IV total between one and two doses) followed by surgery and then adjuvant nivolumab (480 mg IV every four weeks) for up to nine doses. The primary endpoint was recurrence-free survival in all patients (RFS; time from randomization to disease recurrence or death from any cause; investigator assessed). Secondary endpoints were RFS in ccRCC, overall survival, safety, and tolerability. The median age was 61 years. Increased risk of recurrence was defined as clinical T2 disease or higher and/or clinical lymph node disease. Oligometastatic patients were allowed later in an amendment and totaled approximately 4% of patients. Only 71% of patients had confirmed ccRCC. At a median follow up of approximately 30 months, the HR for recurrence-free survival for the nivolumab-plus-surgery group versus the surgery-only group was 0.94 (95% CI, 0.74–1.21; *p* = 0.32). A total of 33% of patients in each arm had recurrence-free survival events (no surgery, not disease-free after surgery, recurrence, and/or death). In the 71% of patients confirmed to have ccRCC via biopsy or surgery, stratified HR for recurrence-free survival was 0.91 (95% CI 0.69–1.22; *p* = 0.54). Finally, post hoc 5-year overall survival was 73% (95% CI 61.1–87.3%) in the nivolumab-plus-surgery group and 81% (72.8–90.4) in the surgery-only group. The HR for overall survival was 1.28 (0.84–1·95; *p* = 0.26). Median overall survival was not reached in either group [21].

As one may infer, the results from the PROSPER trial dampened the momentum for the use of neoadjuvant ICI in the RCC space. However, it is our opinion that certain elements of the trial design may have played a significant role in the overall results. One key element is that clinical staging was utilized for enrollment and approximately 50% of patients had clinical T2 disease. It is well known that clinical staging does not always correlate with pathological staging. Thus, it is likely that this population represented a lower risk of RCC recurrence and therefore limited the benefit of ICI. Another potential limitation of the trial design is the duration of neoadjuvant ICI. A cumulative dose of 480 mg of nivolumab over just 3–4 weeks was given. This is significantly less in dosage and/or duration than multiple positive neoadjuvant immunotherapy clinical trials in other disease sites such as melanoma (e.g., three doses of neoadjuvant pembrolizumab over 9 weeks) [22], non-small cell lung cancer (e.g., four cycles of durvalumab over 12 weeks) [23], and bladder cancer (e.g., four cycles of durvalumab in addition to standard chemotherapy over 12 weeks) [20]. Although not clearly compared in clinical trials, the authors of PROSPER suggest that this relative undertreatment could be a factor in the lack of efficacy found. Third, another noteworthy element of the trial design in PROSPER is that only approximately 70% of patients had confirmed ccRCC. It is now widely accepted that ccRCC can behave in a significantly different fashion compared to non-clear cell RCC (nccRCC). Particularly, multiple nccRCC types are thought to have a “colder” immune microenvironment with decreased immunogenicity and lower mutational burden. For instance, papillary RCC, the most common of the nccRCC types (estimated at 10–15% of all RCCs), was found to have decreased tumor immune infiltrate compared to ccRCC. In addition, only 10% of papillary RCC tumors were found to express PD-L1 [24]. Meanwhile, chromophobe RCC (the second most common nccRCC type) was found to have decreased tumor immune infiltration with significantly decreased CD8+ T-cells that also demonstrated lower immune-checkpoint expression [25]. The biological implications of this data are that nccRCC tumors may not present sufficient neoantigens to prime effective cytotoxic T-cell responses, thus weakening the effect of immune-checkpoint inhibitors. It is important to note that the authors of PROSPER were cognizant of this and did power the study to assess a pre-specified ccRCC population. However, it is our view that future studies should ideally include only ccRCC due to the overall heterogeneity between these types of cancer. Finally, other potential limitations of PROSPER include an open-label design (possibly allowing for more attrition after surgery) and the lack of imaging after neoadjuvant treatment to accurately restage patients.

### 3.3. Current Trials in the Perioperative Immunotherapy Arena

Multiple trials are ongoing in the adjuvant ICI space. At the 2025 European Society for Medical Oncology (ESMO) Annual Meeting, the RAMPART trial was first presented. RAMPART is a phase 3, open-label, multi-arm trial of adjuvant durvalumab monotherapy or in combination with tremelimumab for resected RCC. A total of 750 patients that were post-nephrectomy were randomized in a 3:2:2 ratio to active monitoring vs. durvalumab 1500 mg every four weeks for one year vs. durvalumab 1500 mg every four weeks for one year plus tremelimumab 75 mg for two doses (at day 1 and day 28). The primary endpoint was disease-free survival (DFS). A unique aspect of the trial was that the Leibovich scoring system was used to assess risk of recurrence. The results of the durvalumab monotherapy arm were not available at the time of the presentation. However, preliminary results show that at a median follow up of 36 months, durvalumab–tremelimumab improved DFS compared to active monitoring (HR 0.65, 95% CI 0.45–0.93, *p* = 0.009) in the overall intention-to-treat population. Moreover, what was arguably more significant was that upon closer examination of the data in a pre-specified, pre-powered subgroup analysis, the HR for DFS was even lower in the high-risk population (HR 0.52, 95% CI 0.34–0.80, *p* = 0.002). In contrast, there was no significant benefit in DFS for the intermediate-risk population (HR 1.19, 95% CI 0.61–2.32, *p* = 0.31) [26].

Although the results of PROSPER were not as previously hoped, multiple trials are also ongoing in the neoadjuvant realm. At the 2025 ESMO Annual Meeting, the NESCIO trial was first presented. NESCIO is a phase 2, randomized, non-comparative, open-label, two-stage trial investigating neoadjuvant immunotherapy in intermediate-to-high risk, histologically confirmed, resectable ccRCC. A total of 42 patients were randomized in a 1:1:1 ratio for two doses of nivolumab (anti-PD-1) 360 mg every 3 weeks vs. two doses of ipilimumab 1 mg/kg plus nivolumab 3 mg/kg every 3 weeks vs. two doses of relatlimab (LAG-3 inhibitor) 360 mg plus nivolumab 360 mg every 3 weeks. The primary endpoint was the pathological response rate, and secondary endpoints were event-free and recurrence-free survival. A near pathologically complete response (near-PCR; 1–10% residual tumor) was seen in 14.3% of patients receiving neoadjuvant ipilimumab–nivolumab. A total of 14.3% of patients receiving relatlimab–nivolumab achieved at least a partial pathological response (pPR; 10–50% residual tumor). Both preceding arms thus met their primary endpoints. At a median 22 months of follow up, patients who attained a response had a recurrence-free survival of 100%. This is despite receiving only two doses of immunotherapy over 6 weeks [27]. Although this is a small phase 2 trial, the reported preliminary results are promising.

The NESCIO trial brings up the dilemma of what the medical oncologist should do in terms of adjuvant therapy in the setting of a complete pathological response. Such a perspective is mirrored in the NADINA trial in melanoma, published in 2024. NADINA was a phase 3 trial where 423 patients with resectable, macroscopic stage III melanomas were randomized into two cycles of neoadjuvant ipilimumab plus nivolumab followed by surgery or surgery followed by 12 cycles of adjuvant nivolumab. Patients who had a locally assessed major pathological response (≤10% residual viable tumor) did not receive any adjuvant therapy, whereas those with partial or no response did receive some sort of adjuvant therapy. At a median follow up of 9.9 months, the estimated 12-month event-free survival was 84% (99.9% confidence interval [CI], 73.8 to 94.8) in the neoadjuvant group and 57% (99.9% CI, 45.1 to 72.7) in the adjuvant group. A total of 59% of patients in the neoadjuvant group had a major pathological response. In this group of responders, the estimated 12-month recurrence-free survival was 95.1%, compared to 76% and 57% in those with partial response and non-response, respectively [17]. This trial elegantly demonstrates that one potential benefit of neoadjuvant immunotherapy is the opportunity to risk-stratify further treatment based on the pathological response at surgery.

Other ongoing randomized neoadjuvant trials investigating immunotherapy in non-metastatic ccRCC include perioperative pembrolizumab plus lenvatinib (clinicaltrials.gov ID #NCT04393350) and perioperative toripalimab plus lenvatinib (clinicaltrials.gov ID #NCT06114940). Major prospective trials investigating neoadjuvant and/or adjuvant ICI in RCC are summarized in Table 1 below.

**Table 1:** Major prospective trials investigating neoadjuvant and/or adjuvant ICI in RCC.

Clinical Trial	Experimental Treatment(s)	Control Arm	Primary Endpoint(s)	Key Secondary Endpoint(s)	Summary of Key Findings
**KEYNOTE-564** NCT#03142334	Adjuvant pembrolizumab	Placebo	**DFS:** HR 0.72 (95% CI, 0.59–0.87)	**OS:** HR 0.62 (95% CI, 0.44–0.87), *p* = 0.005	Statistically significant improvement in DFS and OS
**IMmotion-010** NCT#03024996	Adjuvant atezolizumab	Placebo	**DFS** (investigator assessed): HR 0.93 (95% CI, 0.75–1.15), *p* = 0.50	**DFS** (independent review); **OS**	No statistically significant improvement in DFS or OS
**CheckMate-914** NCT#03138512	Adjuvant nivolumab ± ipilimumab	Placebo	**DFS:** PART A: HR 0.92 (95% CI, 0.71–1.19), *p* = 0.53 PART B: HR 0.87 (95% CI, 0.62–1.21), *p* = 0.40	**OS**	No statically significant improvement in DFS or OS
**PROSPER (EA143)** NCT#03055013	Neoadjuvant and adjuvant nivolumab	Surveillance	**RFS:** HR 0.94 (95% CI, 0.74–1.21), *p* = 0.32	**RFS** **in ccRCC, OS**	No improvement in DFS or OS
**RAMPART** NCT#03288532	Adjuvant durvalumab ± tremelimumab	Active monitoring	**DFS:** HR 0.65 (95% CI, 0.45–0.93), *p* = 0.009; **OS:** durvalumab + tremelimumab arm	Not specified	Preliminary improvement in DFS in durvalumab + tremelimumab arm; OS data not available
**NESCIO (phase II trial)** NCT#05148546	Neoadjuvant nivolumab monotherapy vs. ipilimumab + nivolumab vs. relatlimab + nivolumab	N/A	**Pathological response rate** *: 14.3% in ipi + nivo and relatlimab + nivo arms; 7.1% in nivolumab monotherapy	**EFS and RFS**	Both combination immunotherapy arms reached their primary endpoint

* Preliminary data.

### 3.4. Lessons from the Past and Future Directions–Biomarkers, Imaging, and Novel Therapies

To recap, KEYNOTE-564 was the only trial to show the efficacy of adjuvant immunotherapy until RAMPART more recently (OS immature). No trial has yet published the efficacy of neoadjuvant immunotherapy, with the landmark PROSPER trial being the major reference in the literature. However, before ruling out a role for perioperative immunotherapy, one must recognize that significant differences in the trial designs (resulting in wide ranges of risk across populations), suboptimal (e.g., clinical) staging, and great variabilities in the cumulative dosage and/or duration of neoadjuvant and/or adjuvant immunotherapy regimens also exist across the trials. One conclusion that seems increasingly clear is that immunotherapies may be of greatest benefit in reducing ccRCC recurrence in higher-risk populations. This is exemplified by the overall high-risk population and positive result in KEYNOTE-564, and the pre-specified analysis showing demonstrable efficacy in the high-risk subgroup of RAMPART.

How can these lessons be applied? First, one may argue that a more uniform definition of high-risk disease should be applied across trials. A standardized scoring system such as the Leibovich scoring system (utilized in RAMPART) is one potential solution. Another aspect is to increase the precision of the definition of M1 NED (or not include metastatic disease at all). Third, future studies investigating neoadjuvant immunotherapy for a greater duration/dose (e.g., longer duration than 4 weeks) could limit the confounding of undertreatment.

As it pertains to the inaccurate pre-operative clinical staging and neoadjuvant immunotherapy, an ideal solution would be to discover a biomarker that could more accurately diagnose high-risk disease. This biomarker could then hopefully better stratify high-risk patients that would theoretically benefit more from neoadjuvant immunotherapy, as suggested by early data. Clinical trials enriched for these high-risk patients could ultimately prove a role for neoadjuvant treatment. One biomarker that has been studied in a retrospective fashion to better stratify the risk of disease in RCC is Kidney Injury Molecule-1 (KIM-1). KIM-1 is a transmembrane glycoprotein that is overexpressed by kidney cancers (e.g., ccRCC and papillary RCC) originating in the proximal tubule of the kidney. It has been found in the urine and plasma of these patients and is thus minimally invasive [28]. In a matched, case–control study of 162 patients with ccRCC with median follow up of approximately 10 years, higher pre-nephrectomy KIM-1 (pKIM-1) levels were associated with worsened metastasis-free survival (HR 1.29 per unit increase in log pKIM-1, 95% CI 1.10–1.53) and overall survival (OS; HR 1.31 per unit increase in log pKIM-1, 95% CI 1.10–1.54) [29]. Similar trends have been seen in the adjuvant trials of RCC [30]. High serum KIM-1 level was associated with worsened disease-free survival (DFS) and OS in the ASSURE trial, which investigated adjuvant sunitinib, sorafenib, or placebo among patients with high-risk RCC [9]. This phenomenon was also observed in retrospective analyses of adjuvant immunotherapy trials. In CheckMate-914 (adjuvant ipilimumab plus nivolumab) and IMmotion-010 (adjuvant atezolizumab), the highest quartile of pre-treatment serum KIM-1 (compared to other quartiles) in the former and high baseline serum KIM-1 in the latter were both associated with worsened DFS. Interestingly, the subgroup of patients with high KIM-1 who were treated with immunotherapy had a statistically significant improvement in DFS in IMmotion-010 and a non-statistically significant trend toward a DFS benefit in CheckMate-914 [13,14,15]. Further study of KIM-1 has been conducted in the metastatic RCC space, but will not be discussed for the sake of brevity. While these insights are intriguing as they suggest both the potential utility for KIM-1 as a risk stratification tool and possibly as an indicator of immunotherapy response, these results are retrospective and warrant prospective investigation.

Other biomarkers that have shown promise in ccRCC are mucin 1 (MUC1) and its soluble form, cancer antigen 15-3 (CA15-3). In molecular analyses, MUC1 was associated with increased angiogenesis, hypoxia/metabolism regulation, and complement system activation. Particularly, in vitro and in vivo models showed a correlation between MUCI1 overexpression and increased microvascular density. Clinically, patients with non-metastatic ccRCC tumors expressing high levels of MUC1 were found to have poor OS (*p* = 0.01) and PFS (*p* = 0.01). Similarly, RFS was significantly shorter for patients with non-metastatic ccRCC and high levels of CA 15-3 (*p* = 0.002) [31]. These translational and clinical findings highlight the potential of these two biomarkers to serve as prognostic markers to identify aggressive diseases.

In addition to potential biomarkers, an area of increasing interest is whether novel imaging techniques can be used to more accurately perform pre-operative clinical staging of ccRCC patients. According to a single-center, retrospective study of 412 patients, the sensitivity of MRI for renal malignancy was high, but the specificity was low [32]. This is especially true for indeterminate renal masses. Furthermore, FDG PET CT is not recommended for most situations in the diagnosis of renal cell cancer as per NCCN guidelines. This is, in part, because of the physiological excretion of FDG from the kidneys, which decreases the contrast between renal lesions and normal tissue [33]. An imaging modality that is garnering interest in RCC is carbonic-anhydrase IX (CA-IX) PET-CT. CA-IX is a tumor antigen that is highly expressed in ccRCC. Meanwhile, Zr-labeled monoclonal antibody ([^89^Zr]Zr-girentuximab) has high affinity for CA-IX. ZIRCON was a prospective, phase 3, multi-center trial in which 332 patients with small (≤7 cm) renal lesions were given one dose of [^89^Zr]Zr-girentuximab intravenously followed by abdominal PET–CT imaging five days (±2 days) later. Patients underwent surgery within 90 days. At analysis, the mean sensitivity and specificity for ccRCC was 85.5% (95% CI 81.5–89.6) and 87.0% (95% CI 81.0–93·1), respectively. The safety profile was also found to be favorable [34]. Finally, in the latest literature, radiolabeled peptides targeting CA-IX may have potential benefits over monoclonal antibodies targeting CA-IX such as Zr-labeled girentuximab. One example is Ga-cyclic peptides such as [^68^Ga]Ga-DPI-4452 PET/CT. Compared with antibody PET/CT imaging, ^68^Ga peptides have on-site on-demand production, rapid tumor uptake, and rapid plasma clearance that results in optimal imaging times of about 60 min. This contrasts with 5 days for Zr-labeled girentuximab. [^68^Ga]Ga-DPI-4452 PET/CT has a lower radiation dose and is logistically more feasible as everything can theoretically be done in a single clinic visit [35]. Whether antibody or peptide, these new radiotracers targeting CA-IX are non-invasive modalities that have the potential to cause a paradigm shift in clinical practice by more accurately staging pre-operative patients and possibly better stratifying risk in ccRCC. Of note, an interesting research question is whether combining new biomarkers such as KIM-1 with these new imaging modalities could yield additive or synergistic positive effects to the clinical diagnosis and management of ccRCC.

Expanding further into potential paradigm-shifting methods of staging, artificial intelligence (AI) has generally taken the world by storm. Its uses in radiology and pathology are known as radiomics and pathomics, respectively [36]. In RCC specifically, radiomics has shown excellent diagnostic accuracy for the detection of RCC, the ability to distinguish clear cell vs. non-clear cell subtypes, and it can even predict the nuclear grade [37]. Radiogenomics, a subset of computational radiology, investigates the association of radiomic and quantitative features with gene mutations and gene expression-based molecular signatures. For instance, one study found an association between the level of MRI contrast enhancement and angiogenic gene expression (high enhancement) or immune-related gene expression signatures (low enhancement) in ccRCC [38]. Given the challenges of tumor heterogeneity with traditional tumor biomarkers, radiogenomics presents an exciting potential alternative as an imaging surrogate biomarker.

Finally, as we seek to establish the truth as it pertains to perioperative immunotherapy in RCC, novel therapies that seek to augment immunotherapy are also being studied. One novel concept is whether neoantigen-targeting personalized cancer vaccines (PCVs) with or without immunotherapy can be administered in an adjuvant fashion to reduce recurrence in high-risk ccRCC. The hypothesis is that targeting neoantigens such as RCC driver mutations (e.g., VHL, PBRM1, BAP1, KDM5C, PIK3CA) leads to immune system recognition of tumors and a greater clinical response. In a phase 1 trial investigating this concept with or without ipilimumab in patients with high-risk, resected ccRCC, none of the nine patients experienced RCC recurrence at a median follow up of 40 months [39]. This concept has now been advanced to a phase 2 trial studying adjuvant pembrolizumab with or without the personalized vaccine called V940 (mRNA-4157; NCT06307431). Finally, in the relapsed/refractory setting, trials are now studying chimeric antigen receptor T-cell (CAR-T) therapies in ccRCC. One such phase 1 trial of 16 patients with relapsed/refractory ccRCC yielded an 81% disease control rate and no patients experienced dose-level toxicities [40]. CAR-T has now entered phase 2 trials in the relapsed/refractory setting [41], and it remains to be seen whether this will be adopted in the adjuvant arena.

**Figure 1:**
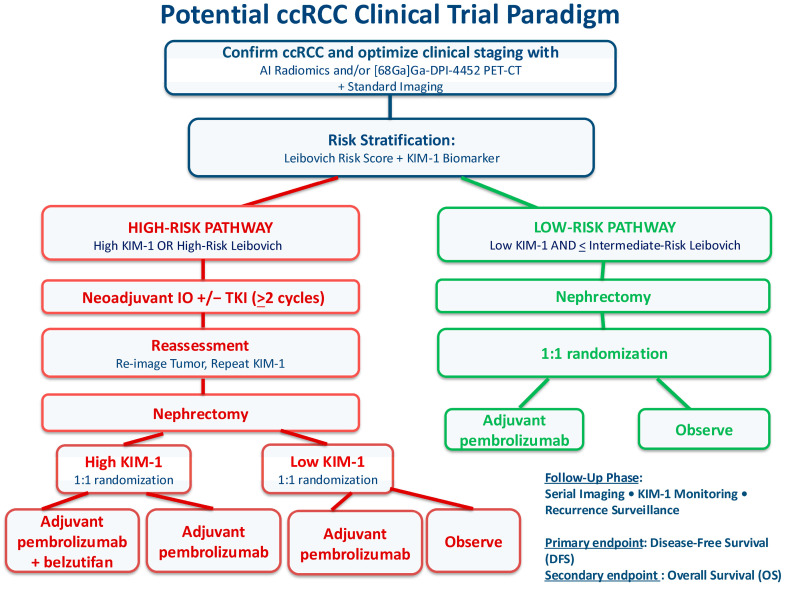
Potential clinical trial paradigm incorporating lessons from prior clinical trials.

An example of a novel clinical trial paradigm can be seen in Figure 1. The purpose of creating and including this paradigm is simply to reinforce the topics discussed in this article and illustrate one way to apply them critically. It is not meant to be viewed as a proposal for a clinical trial. The paradigm aims to highlight how one may incorporate the lessons learned from prior trials, leverage new technologies, and further investigate belzutifan (in addition to immunotherapy) to optimize both the diagnosis and management of high-risk ccRCC. We hope that it can help generate critical discussion about the application of the lessons learned in the ccRCC space.

## 4. Conclusions

Despite the results of the PROSPER trial, there is ample evidence to argue that neoadjuvant treatment is worthy of further investigation in RCC. A review of the literature suggests that the foundational challenge is being able to accurately stratify risk to offer therapy to the appropriate patients. This may require an unconventional approach, and an overall paradigm shift in the workup of RCC. Thankfully, a wealth of novel technologies and treatments is now at our disposal to hopefully help move the needle forward. In a field of relative stagnation compared to other cancers, the future of RCC is bright.

## Data Availability

New data were not created or analyzed in this study. Data sharing is not applicable to this article.

## Abbreviations

The following abbreviations are used in this manuscript:

RCC	Renal cell carcinoma
ccRCC	Clear cell renal cell carcinoma
ICI	Immune-checkpoint inhibition
DFS	Disease-free survival
OS	Overall survival
VEGF-TKI	Vascular endothelial growth factor tyrosine kinase inhibitor
CTLA-4	Cytotoxic T-lymphocyte antigen 4
PD-1	Programmed cell death protein 1
PD-L1	Programmed death-ligand 1
M1 NED	Metastatic 1, no evidence of disease
AI	Artificial intelligence
CAR-T	Chimeric antigen receptor T-cell therapy
